# Self-Assembled Ag Nanocomposites into Ultra-Sensitive and Reproducible Large-Area SERS-Active Opaque Substrates

**DOI:** 10.3390/nano11082055

**Published:** 2021-08-12

**Authors:** Abeer Fahes, Aotmane En Naciri, Mohammad Navvabpour, Safi Jradi, Suzanna Akil

**Affiliations:** 1 LCP-A2MC, Jean Barriol Institute, Lorraine University, 1 Arago Avenue, 57070 Metz, France; abeer.fahes@univ-lorraine.fr (A.F.); aotmane.en-naciri@univ-lorraine.fr (A.E.N.); 2 Light, Nanomaterials, Nanotechnologies (L2n) Laboratory, CNRS ERL 7004, University of Technology of Troyes, 12 Rue Marie Curie, 10004 Troyes, France; mohammad.navvabpour@utt.fr (M.N.); safi.jradi@utt.fr (S.J.)

**Keywords:** SERS, nanosensors, plasmonic nanoparticles, nanofabrication, VIPS, self-assembly

## Abstract

This work describes a novel, one-shot strategy to fabricate ultrasensitive SERS sensors based on silver/poly(methyl methacrylate) (PMMA) nanocomposites. Upon spin coating of a dispersion of PMMA and silver precursor on N-doped silicon substrate, closely separated silver nanoparticles were self-assembled into uniform nanospheres. As a result, a thin hydrophobic PMMA layer embedded with Ag nanoparticles (AgNPs) was obtained on the whole silicon substrate. Consequently, a large-scale, reproducible SERS platform was produced through a rapid, simple, low-cost, and high-throughput technology. In addition, reproducible SERS features and high SERS enhancement factors were determined (SEF ~10^15^). This finding matches the highest SEF reported in literature to date (10^14^) for silver aggregates. The potential and novelty of this synthesis is that no reducing agent or copolymer was used, nor was any preliminary functionalization of the surface carried out. In addition, the AgNPs were fabricated directly on the substrate’s surface; consequently, there was no need for polymer etching. Then, the synthetic method was successfully applied to prepare opaque SERS platforms. Opaque surfaces are needed in photonic devices because of the absence of secondary back reflection, which makes optical analysis and applications easier.

## 1. Introduction

Surface-enhanced Raman spectroscopy (SERS), which integrates high levels of sensitivity with spectroscopic precision, provides huge enhancements to Raman signals of trace detection levels of chemical and biological molecules adsorbed on metal surfaces [1,2,3]. The measured Raman signal enhancement in SERS shows impressive enhancement factors, up to 14–15 orders of magnitude, thus enabling the ultrasensitive identification of even single molecules [4,5].

The ultrasensitivity of metallic-nanoparticle-based SERS substrates is usually linked to the high number of hotspots formed within the small interspaced gaps between the constituent nanostructures [6]. Moreover, the enhancement of the SERS signal is highly dependent on the plasmonic resonance frequency of synthesized nanoparticles (NPs) [7]. Generally, as a prerequisite for a maximum intensity SERS signal, there must be a close match between the wavelength (λ) of the plasmon resonance peak of the NPs and the Raman excitation wavelength [7]. Therefore, from an application standpoint, it is crucial to manipulate the interparticle spacings between metallic nanostructures and extract their optical properties.

Recently, a growing body of literature has extensively studied and evaluated several self-assembly approaches in SERS studies [8,9,10]. Currently, different techniques are being used to arrange self-assembled AgNPs over a large area, thanks to polymers [11,12,13,14,15], block copolymers [16,17,18], dendrimers [19], proteins [20], and deoxyribonucleic acid (DNA) molecules [21,22] acting as matrices for inducing ordering and anisotropic orientation on surfaces, and for controlling the number of constituent particles and their separation. [23,24,25,26].

To overcome the serious shortcomings—including uniformity and unsatisfactory reproducibility—in SERS performance, as recognized by the self-assembly approaches [26,27,28,29], it is essential to establish a rational and facile method for the fabrication of AgNPs substrates with controllable nanogaps. Additionally, it is highly desirable to enhance the quality of synthesized nanostructures by producing free-standing, flexible, and large-scale SERS substrates with uniform, stable, reproducible, and highly sensitive SERS signals. In fact, as far as we know, there are still no exhaustive studies applied to synthesize AgNPs via a surface-based strategy.

Herein, in order to reach this ultimate goal, we fabricated AgNPs on a surface via a convenient chemical synthetic route known as vapor-induced phase separation (VIPS). This highly versatile and unprecedented approach was developed recently by our laboratory team for the fabrication of precisely shaped gold nanoparticles embedded in a poly(methyl methacrylate) (PMMA) layer [30]. VIPS is considered to be a powerful and simple route because it provides excellent control of the structural properties of NPs. It offers compelling evidence for producing efficient SERS platforms with controlled size, shape, and interparticle gap distances. The success of this approach is obvious in fabricating nanostructures without demanding long processing times, tedious steps, high overheads, high temperatures, or the use of toxic chemicals, reducing agents, and surfactants. Moreover, there is no need for either functionalizing the surface or etching of polymer film. Nevertheless, the VIPS approach is well suited for producing controlled nanogaps below 10 nm.

To accomplish this approach, layers of AgNO_3_/PMMA dispersions were deposited on a conducting Si wafer in order to allow the spontaneous formation of large-area SERS-active substrates of AgNPs. To be precise, we carried out a parametric study dealing with the influence of different experimental parameters on the optical and structural properties of AgNPs, such as concentration of Ag precursor and spin-coating speeds. Studies were executed under the conditions described in the Materials and Methods section. Changes in the morphological features of the substrates were identified via scanning electron microscopic (SEM) characterizations. The optical properties of synthesized AgNP substrates were monitored through micro-extinction and ellipsometric optical measurements. Ultimately, we assured the effectiveness of AgNPs substrates in SERS applications by using trans-1,2-4 (bipyridyl) ethylene (BPE) as the Raman probe molecule.

## 2. Materials and Methods

### 2.1. Synthetic Approach

**Figure 1:**
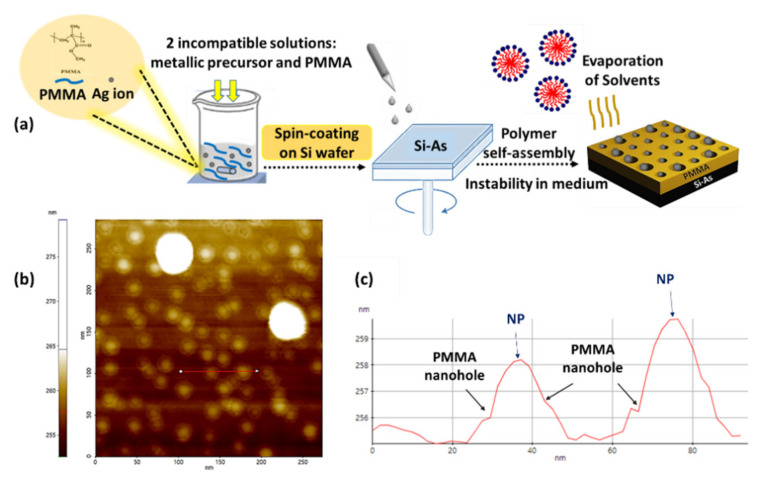
(**a**) Schematic representation of the VIPS approach, showing the growth of two different diameters of AgNPs into PMMA nanoholes attached with (**b**) an AFM image, and (**c**) its corresponding line -scan profile.

The experimental procedure bears a close resemblance to the one proposed previously by our group [30,31,32,33]. The main principle of this technique relies on the self-assembly of thin-layer poly(methyl methacrylate, PMMA) (Sigma-Aldrich, Kappelweg 1, Schn., Germany) into nanoholes, which are used as synthesis reactors for metallic NPs (MNPs). More details about this synthesis and mechanism are given in our previous papers [30,31,32,33]. Specifically, we aimed to extend the self-assembly approach to a large variety of MNPs with different structural properties. As a brief summary, the strategy was based on a nanophase separation between two thermodynamically incompatible solutions. Upon depositing the mixture by spin-coating on conductive substrates, a thermodynamic instability was prevalent. Here, N-doped silicon substrate was used to allow spontaneous reduction of the silver precursor. Silicon plays a crucial role in promoting electrons to Ag^+^ and then producing AgNPs, which prevents the use of any external reducing agent, so that our samples are obtained by a one-shot procedure. This arises due to the fact that metallic salts are strongly dissolved in alcohol, which is a non-solvent of PMMA. Consequently, two different sizes of micelles containing M^n+^, ethanol, and acetone were distributed on the substrate surface. These micelles exploded after the evaporation of the solvents, thus leading to the formation of PMMA nanoholes containing two different average diameters of metallic salts due to the complete fitting of NPs into nanoholes. Accordingly, the AgNO_3_/PMMA dispersion was fabricated by mixing two incompatible solutions, i.e., PMMA/acetone (C = 30 g/L) and AgNO_3_/ethanol. Afterwards, a clean silicon wafer was coated with a monolayer of silver solution in order to shield the formation of monodisperse AgNPs on the surface. Drops of silver solution with different concentrations (10, 20, 30, and 40 mM) were spread onto the silicon substrates at different spinning speeds: 3000, 5000, and 7000 rpm. All of the samples were carefully prepared using the following spin-coating parameters: (time: 30 s, acceleration: 3000 rpm s^−1^). Generally, to obtain a homogeneous layer, we must choose an acceleration value lower than the speed. Otherwise, we cannot easily reproduce the same thickness for samples prepared using the same conditions. Next, these samples were replicated under stable conditions to check their reproducibility. Figure 1 illustrates the scheme for the fabrication of AgNPs inside PMMA nanoholes via a surface-based strategy.

### 2.2. Characterizations

Atomic force microscopy (AFM): AFM measurements were performed using a Park Systems NX10 (ParkSystems, Orsay, France) operating in tapping mode. Depending on the analyzed samples, this technique can easily be adapted to suit all requirements. Samples were simply placed on the XY scanner with high orthogonality, and then imaged by using pre-mounted super sharp (SSS-NCHR) probes (nanosensors) with a high mechanical Q factor for high sensitivity. The probes offer unique features, such as: aspect ratio at 200 nm from the tip apex in the order of 4:1, half-cone angle at 200 nm from apex <10°, resonant frequency = 330 kHz, spring constant = 42 N/m, and tip radius of curvature <5 nm.

Scanning electron microscopy (SEM): The instrument employed in collecting SEM images was a HITACHI SU8030 SEMFEG (Carl Zeiss, Oberkochen, Germany), which was manipulated in a secondary electron imaging mode at an accelerating voltage = –15 kV, magnification = 30–250 K, working distance = 8200–9100 µm, and emission current = 7000–15,600 nA. The samples were coated with a 5-nm Pt/Pd metal layer.

Micro-extinction spectra (µext): Measurements were taken using a custom-built BX51 Olympus optical microscope (LORDIL, Nancy, France). The system was fully integrated with SpectraWiz (StellerNet, Tampa, FL, USA) software for recording the optical spectra of samples in the Vis region (400–800 nm). An incident beam from a halogen lamp (size ~10 μm) was localized at normal incidence onto the substrates during all measurements. For opaque substrates, this setup was managed in reflection mode in order to extract the percentage reflection at pixel n using the current sample, reference (silicon wafer), and dark datasets:

(1) $$R_{n}=(({\mathrm{sample}}_{n}-{\;\mathrm{dark}}_{n})/({\mathrm{ref}}_{n}-{\;\mathrm{dark}}_{n}))\times 100$$

where sample, ref, and dark are respective photon energies.

Average data were acquired for a minimum of 6 regions per sample in order to check their homogeneity and reproducibility.

Ellipsometric measurements: The change in polarized light upon light reflection on a sample was measured using a phase-modulated ellipsometer (UVISEL, HORIBA Jobin Yvon SAS, Longjumeau, France) in the 200–1800 nm spectral range. To ensure a high sensitivity of the measurements, an incidence angle of 70° was chosen. The spot size of the used light beam was typically 1000 µm^2^. This setup directly measures the amplitude ratio (Ψ) and phase difference (Δ) angles between p- and s-polarized light. In general, after measurement of absolute values of psi and delta, construction of an optical model is required for data analysis. From modelling, physical properties including the optical constants, absorption coefficients, and film thicknesses of samples can be extracted. Data analysis and modelling were performed using DeltaPsi2 software from Horiba Scientific.

Surface-enhanced Raman scattering (SERS): SERS measurements were performed on a Dilor Jobin-Yvon Spex instrument (HORIBA Jobin Yvon SAS, Longjumeau, France) from Horiba with a 632.8 nm laser and CCD detection. Both excitation and collection were conducted through a long-distance 50-x. Results were collected on a 10-µL bipyridyl ethylene (BPE) droplet deposited on AgNP substrate. The measurements were made after the evaporation of water from the drop in order to obtain a higher signal/noise ratio. We used a laser power of 5 mW and an acquisition time of 5 seconds. It is noteworthy that it was not possible to obtain the same spectra (peaks) in the same measurement conditions (time, laser power, pinhole, etc.) for RAMAN and SERS. All Raman analyses were performed with 10^−2^ M of BPE. Therefore, we increased the concentration of BPE so as to be able to compare between the spectra, and then to calculate an enhancement factor. In order to get an idea about the reproducibility of the results, five experiments were conducted at different parts of the same substrate. After the acquisition of several measurements, an average curve was calculated for each substrate SERS signal at a given BPE concentration.

## 3. Results

### 3.1. Adjusting the Optical and Structural Properties of Ag Nanoparticles (AgNPs)

In particular, the promising functions of AgNPs as nanosensors can be optimized through the adjustment of diverse factors, including the concentration of the metal precursor, or the spin-coating speed. This section focuses on the experimental parameters influencing the size distributions, shape, and density of NPs on the surface, the thickness and sizes of the PMMA nanoholes, and the gap distances between the constituent AgNPs. Here, to achieve the best SERS performances, we sought the experimental conditions that would produce an ultrahigh yield of AgNPs with precisely controlled sizes and small gap distances. It is widely known that the strength of the SERS signal of the adsorbed molecules is highly promoted, as the NPs are denser on the substrate, with small interspaced gaps [34].

#### 3.1.1. Impact of Spin-Coating Speed

To further understand the role of spin-coating speed in controlling the structural properties of AgNPs, a set of substrates was fabricated at different speeds, while keeping all other experimental parameters unaltered. Adjusting spin-coating speed is one of the main parameters that can control the density, size distribution, growth rate of formation, and gap distances between NPs. In general, 40 mM was considered to be a critical concentration of silver precursor, since it yields a surface full of high-density AgNPs. It is relevant to note that the maximum concentration reached with Ag precursor was 40 mM. Increasing the concentration of Ag/ethanol beyond this value caused the formation of PMMA aggregates inside the solution. Thus, it can be conceivably hypothesized that repulsive interactions between both Ag and PMMA solutions should be modulated to be relatively weak in order to prevent the phase separation of the whole dispersions.

**Figure 2:**
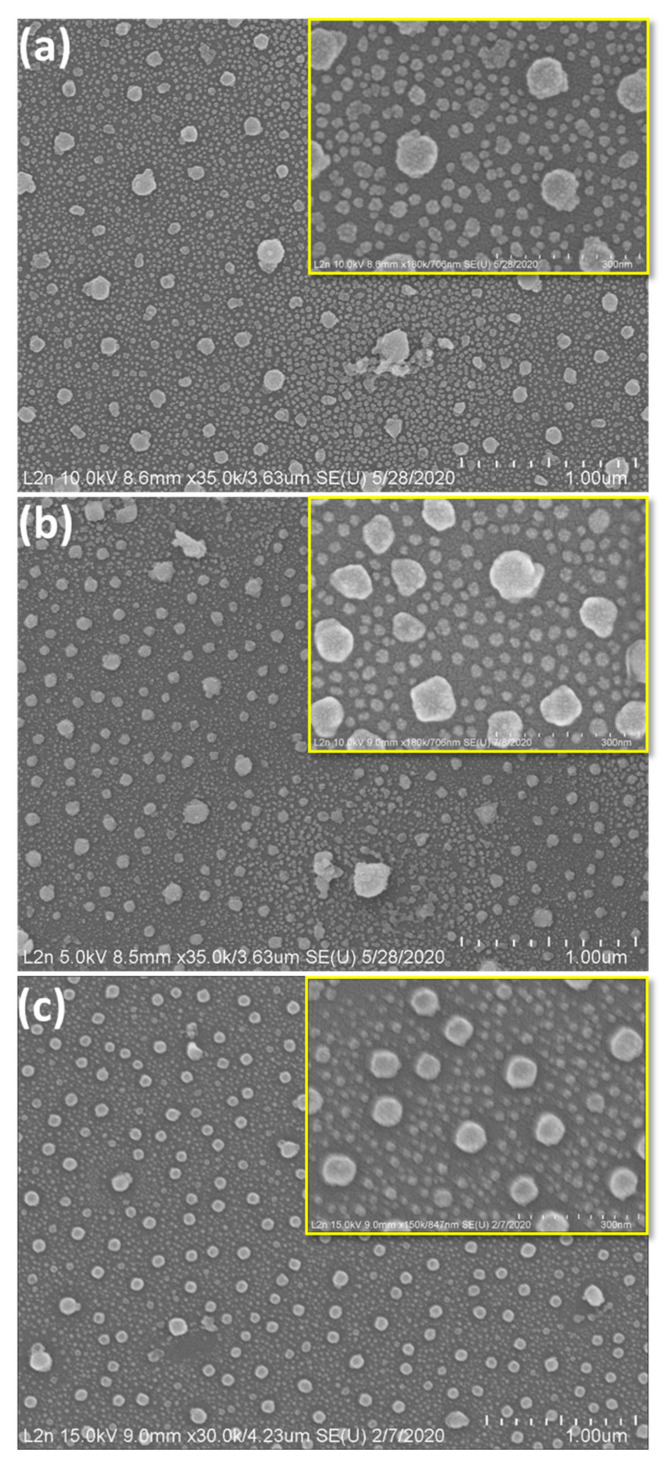
SEM images of 40-mM Ag/PMMA samples fabricated at different spin-coating speeds: (**a**) 3000; (**b**) 5000; and (**c**) 7000 rpm. All insets are the corresponding magnified SEM images at 300 nm.

As illustrated in Figure 2, SEM images reveal that polydispersity in size and shape decreases with increasing speed.

The increase in speed noticeably evolved the morphology of AgNPs from high-index faceted random shapes into nearly isotropic spherical shapes with a high homogeneity over the whole surface. It is noteworthy that we did not aim to produce only spherical nanoparticles, since SERS enhancement is greater with anisotropic nanostructures. To be precise, we wanted to prepare AgNPs using the VIPS strategy that we had mainly developed for gold before now. In a future work, we aim to produce other morphologies. This study requires much more experimentation and investigation of the synthetic method. The most striking observation at 7000 rpm is that all small AgNPs, with an average diameter of about 16 nm, are nearly uniform, monodisperse, spherical, highly dispersed, and organized on the substrate surface with very small interparticle separation distances. Moreover, large AgNPs of average diameter ~74 nm display regular nanoscale patterning features, with gap sizes only a few nanometers apart (<10 nm). Both size regimes of AgNPs exhibit narrow size distributions, with an exquisite control over the size.

The findings at 7000 rpm can lead to important implications for SERS applications. The synthesized substrates, with a high density of hotspots, can be exploited as efficient nanostructures for yielding strong SERS signals from single molecules. In addition, they can be easily scaled for large-scale production due to the greater proportion of different controlled diameters of AgNPs on the surface. Impressively, SERS applications can be readily tuned at variable optical ranges.

**Figure 3:**
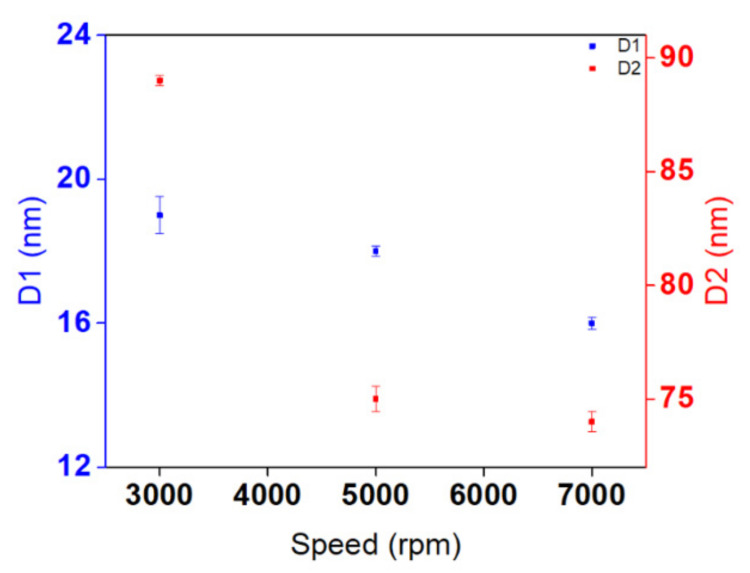
Average diameters (D1 and D2) of AgNPs with their correlated error bars versus spin-coating speed.

**Figure A1:**
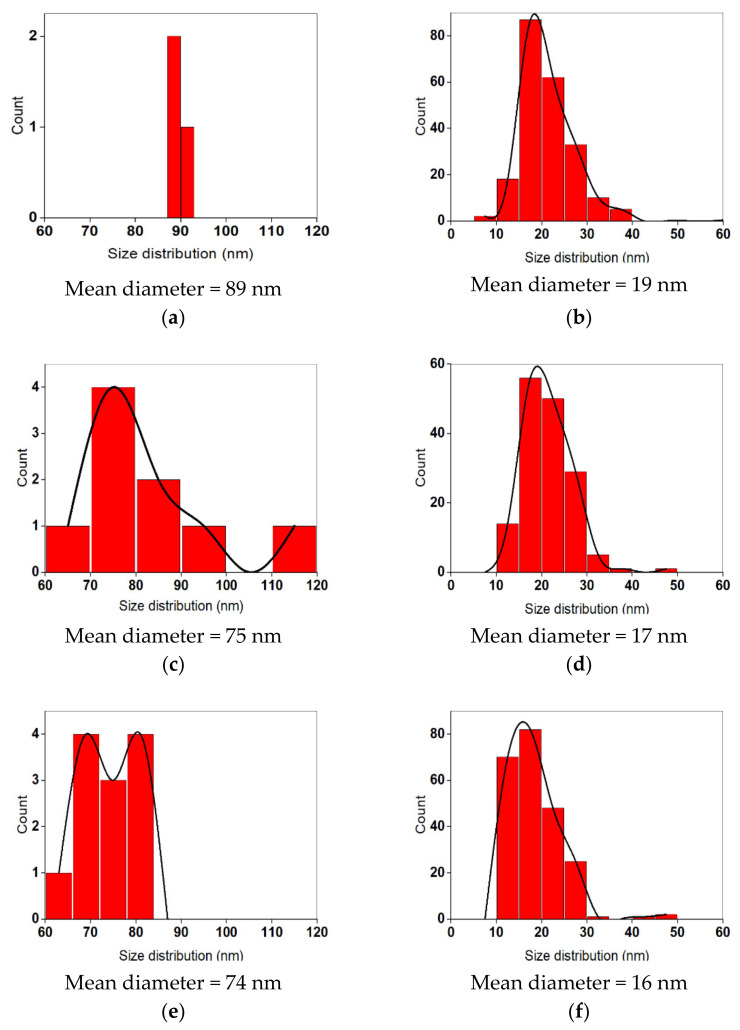
Size distribution histograms for the determination of average diameters (D1 and D2) of AgNPs prepared at different spin-coating speeds: (**a**,**b**) 3000; (**c**,**d**) 5000; and (**e**,**f**) 7000 rpm.

To further investigate the average diameter of AgNPs at different speeds, size distribution measurements were performed (Figure A1). Average diameter changes are presented in Figure 3. Mainly, two different average diameters of AgNPs dominate the entire surfaces at all speeds. By increasing the spin-coating speed from 3000 to 7000 rpm, the diameter of both AgNPs decreases slightly, and then approaches a constant value when exceeding a speed of 5000 rpm.

Spin-coating speed plays an important role in the structuring mechanism, and has a direct influence on the size and distribution of PMMA nanoholes as well as the organization of MNPs inside the holes. In the initial stage of the process, during the deposition of the mixture on the substrate surface, different solvents will start to evaporate according to their volatility and compatibility with the rest of the mixture. This would be the origin of a nanophase separation, which is manifested by the appearance of micelles whose size is dependent on spin-coating speed. At higher spin-coating speeds, the micelles burst rapidly, leaving behind PMMA nanoholes with narrow size distributions. It can be reasonably assumed that these micelles have insufficient time to coalesce into larger micelles; as a consequence, small NPs are obtained, with high density.

**Figure 4:**
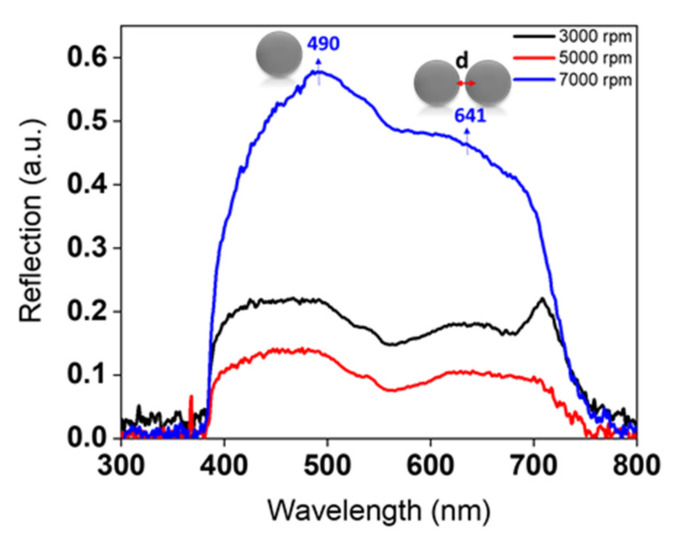
Reflection spectrum of AgNPs/PMMA nanocomposites prepared with different spin-coating speeds. The spectra were extracted using a bright-field optical microscope.

**Figure A2:**
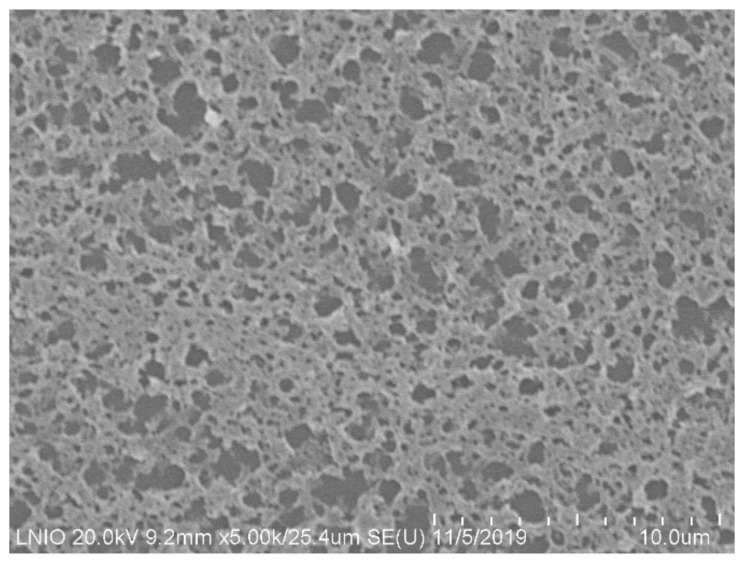
SEM image showing the non-continuous structure of the PMMA/acetone layer.

The optical characteristics of AgNPs assembled into a PMMA matrix are highlighted in Figure 4. Using a micro-extinction optical microscope, we were unable to clearly observe well-defined and standard plasmonic peaks for AgNPs. This is not particularly surprising, given the fact that determining absorption and extinction cross-sections from a reflection spectrum is highly complicated. The foremost cause of this difficulty is due to the deposition of nanostructures on solid, opaque substrates. It is essential to consider that substrates supporting nanostructures can have significant effects on the nanostructures’ LSPR (localized surface plasmon resonance) and near-field distributions [35]. The combination of the halogen lamp’s spectral signature with the corresponding plasmonic bands of the nanostructures is also evidence of the difficulty of collecting optical responses using this setup. Another possible explanation is that the PMMA/acetone surface layer is rough, nanoporous, non-continuous, and anti-reflective [36,37] (Figure A2). The reflection from the porosity of the PMMA nanoholes contributes greatly to the responses of the AgNPs. To date, no suitable method has been detected for removing the PMMA from the surface layer without any disturbance to the structuring. Several trials for removing the PMMA were initiated in order to promote the development of well-defined optical properties of AgNPs using a micro-extinction setup. Unfortunately, all of our attempts failed, since removing the PMMA layer impedes the availability of AgNPs in high quantities on the surface.

The existence of PMMA plays a vital role in the synthetic mechanism. The PMMA layer, acting as a coating support for the NPs, can protect AgNPs from extraneous chemical and physical changes by reducing their reactivity. In a previous paper by our group [23], the SERS enhancement factor was totally diminished upon removing the PMMA, thus indicating its importance in fulfilling high SERS nanofocusing and enhancement due to its hydrophobic properties. Crucially, PMMA acts as a stabilizing agent to prevent aggregation in samples. In addition, it controls the size and shape of NPs, the kinetics of growth, and the diffusion of atoms.

**Figure 5:**
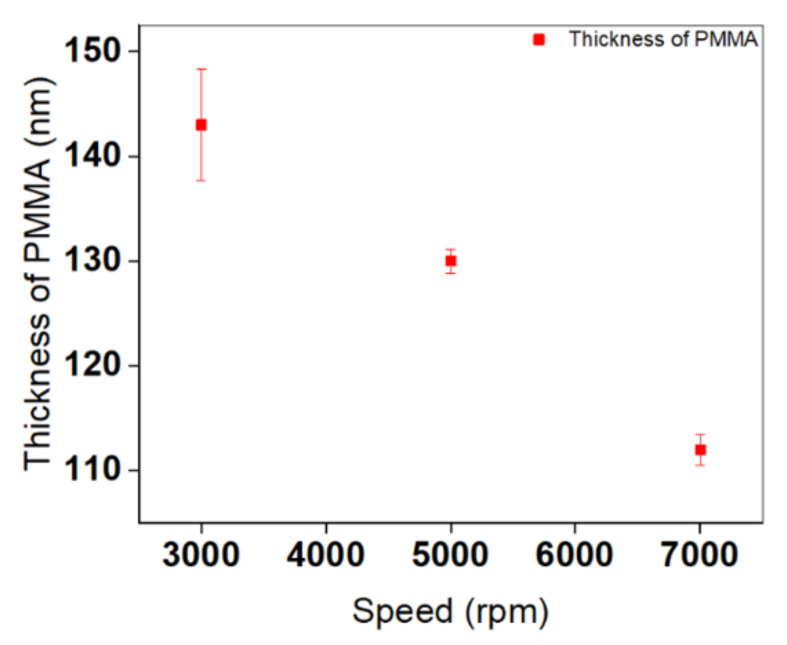
The influence of spin-coating speeds on the thickness of deposited PMMA layers when metal is introduced. Error bars are also displayed in this plot. Measurements of thickness values are extracted using an AFM technique via a line-scratching method.

As shown in Figure 4, the intensity of the peaks shows a remarkable increase at high spin-coating speeds, with an enhancement in magnitude and sharpness. Two optical modes appear at all speeds: one corresponds to the optical response of individual, large AgNPs, and the other to a coupling phenomenon between two closely separated large AgNPs. More details on the attribution of peaks can be found in the “Impact of Concentration of Ag Precursor” section. Thickness-dependent reflected optical spectra of AgNPs are demonstrated from these measurements. As the thickness of the PMMA layer increases, the reflection from AgNPs decreases. In order to acquire evident plasmonic peaks from samples, the layers should be thin enough for the light to penetrate through. This is why it was difficult to detect the optical response clearly at lower speeds—i.e., 3000 and 5000 rpm—due to their high thicknesses, anisotropic geometrical features, and polydispersity, as highlighted in Figure 2 and Figure 5. A clear trend in controlling the thickness of the PMMA is mentioned in Figure 5. The thickness of the resultant PMMA film is tuned from 143 to 112 nm by varying the spin-coating speed, and a thinner layer of PMMA is reproduced well for the substrate prepared at 7000 rpm. Note that a scratch was made in our samples in order to expose the Si wafer and achieve an accurate measurement of the depth of the holes. This was done by means of an AFM technique to predict the overall thickness of the PMMA.

Taken as a whole, speed significantly affects the thickness of the PMMA nanoholes, as well as the shape and monodispersity of the synthesized substrates. However, no noteworthy differences were found in the number of assembled AgNPs, their average diameters, or their gap distances. For this reason, further experimental investigations are needed in order to estimate significant differences in the structural and optical properties of AgNPs, as illustrated in the next section. The prospect of engineering the structural properties of Ag nanostructures and manipulating their plasmonic properties through spectral shifts serves as a major aim for developing good nanosensors in a broad spectral range.

#### 3.1.2. Impact of Concentration of Ag Precursor

**Figure 6:**
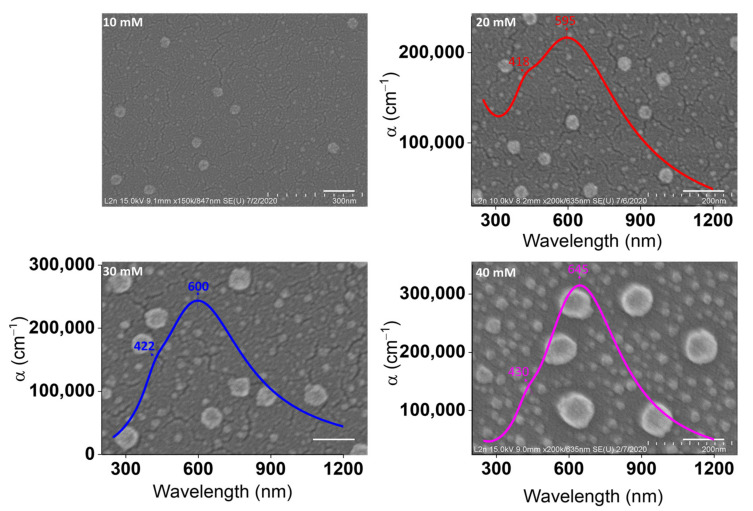
SEM images of Ag samples obtained by varying the concentration of Ag precursor. The scale bar is 100 nm. The insets show the absorption coefficient (α) of AgNPs/PMMA pre-synthesized substrates at each concentration.

To adequately emphasize the effect of concentration, four samples were prepared using the same spin-coating speed: 7000 rpm. Returning to the discussions posed concerning the effects of speed, it is now possible to state that 7000 rpm is the optimal speed. The concentration of AgNO_3_/ethanol varied from 10 to 40 mM. Figure 6 presents the SEM images of the corresponding AgNP monolayers deposited onto the Si substrates at various concentrations. These images provide a visual analysis of the differences in the structural properties of the AgNP substrates. Few particles exist at low concentrations (10 and 20 mM), whereas an excessive number of closely separated particles is dominant at high concentrations (30 and 40 mM). Most of the particles at low concentrations are individually isolated from one another with high distances. On the other hand, at higher concentrations, the increase in the density of NPs leads to a decrease in interparticle separation distances. This study further considers the feasibility of producing AgNPs with interparticle spacings of less than 10 nm.

**Figure A3:**
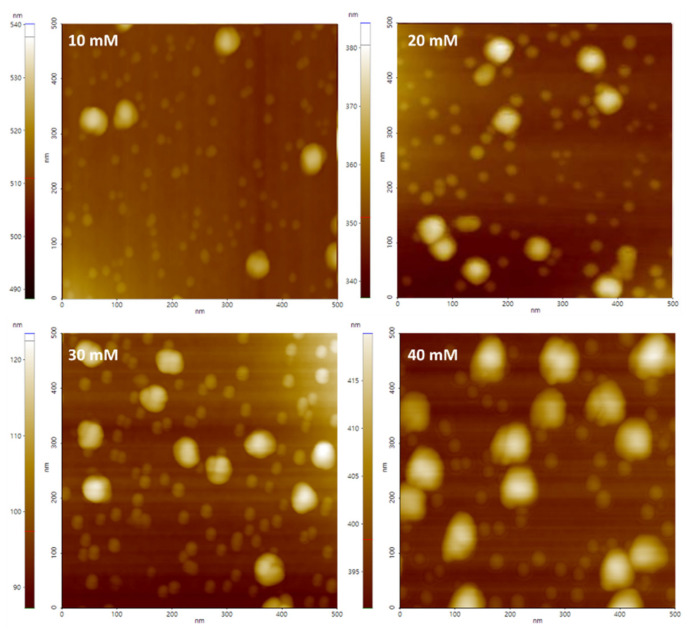
Two-dimensional tapping mode AFM images (500 × 500 nm) for Ag samples prepared at different concentrations.

Figure A3 confirms that the AFM findings are consistent with previous SEM observations. The AFM technique clearly shows its potential in distinguishing small AgNPs at low concentrations (10 and 20 mM) due to the small radius of curvature used for the tip (<5 nm).

**Figure A4:**
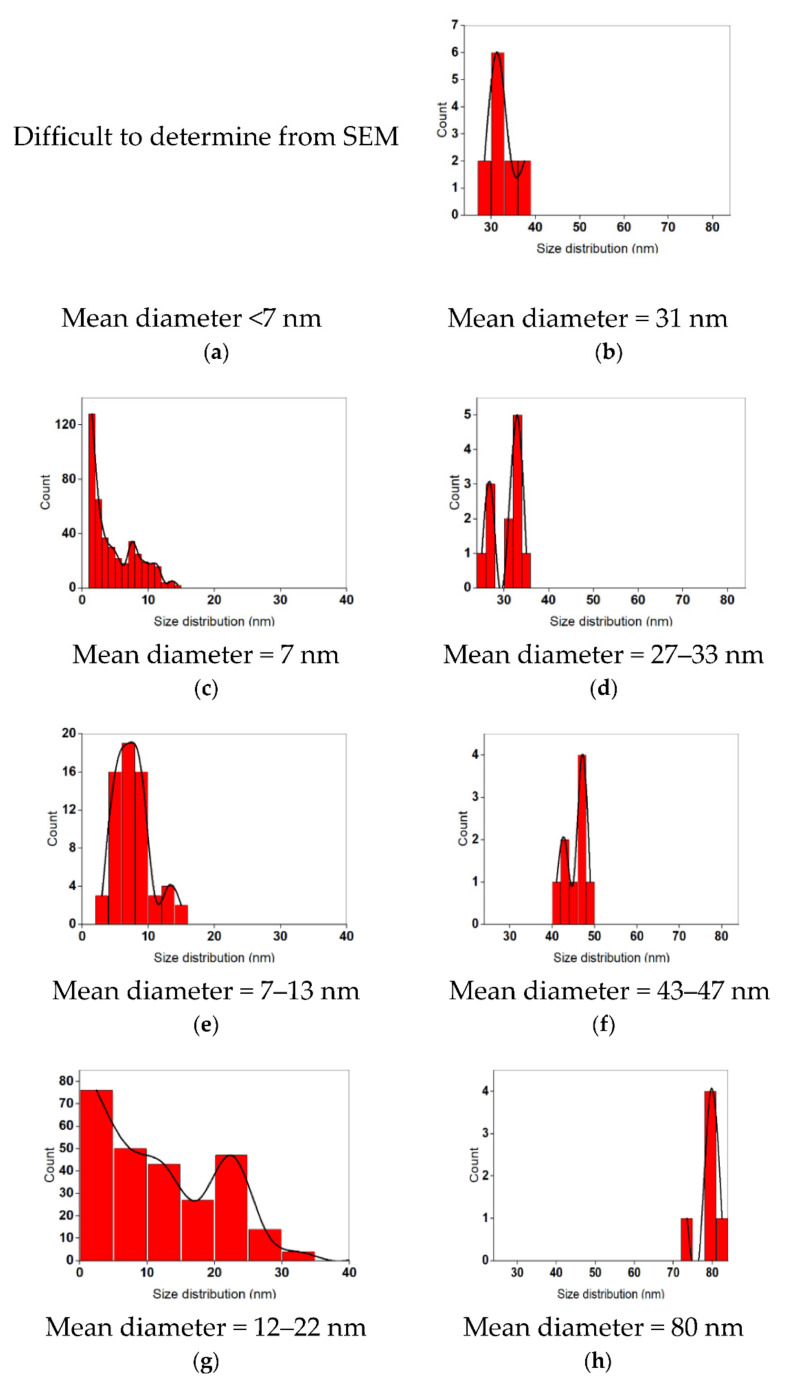
Size distribution histograms for the determination of average diameters (D1 and D2) of AgNPs prepared at different concentrations of Ag precursor: (**a**,**b**) 10; (**c**,**d**) 20; (**e**,**f**) 30; and (**g**,**h**) 40 mM.

The average diameters characterizing the assembled AgNPs are summarized in Table 1. Average diameters are calculated from size distribution histograms at all concentrations (Figure A4). Typically, two different average diameters of AgNPs are dominant at each substrate surface. By increasing the concentration of Ag, the average diameters of AgNPs increase, and reach their highest at 40 mM. This happened because, in some regions, the resulting small AgNPs participate in a subsequent coalescence and growth into larger spheres.

Changing the concentration of Ag precursor can primarily affect the particles’ quality, and their efficiency of self-assembly. When the concentration is too low, thermodynamic instability will be caused, and the self-assembly will be restricted at the early stage due to the aggregation of PMMA particles. Consequently, it is important to reach the optimal Ag concentration in order preserve the self-assembly in medium. This can be achieved by using a concentration beyond 20 mM, which leads to the auto-organization of AgNPs on the surface, with well-ordered assembly and good dispersibility.

**Figure 7:**
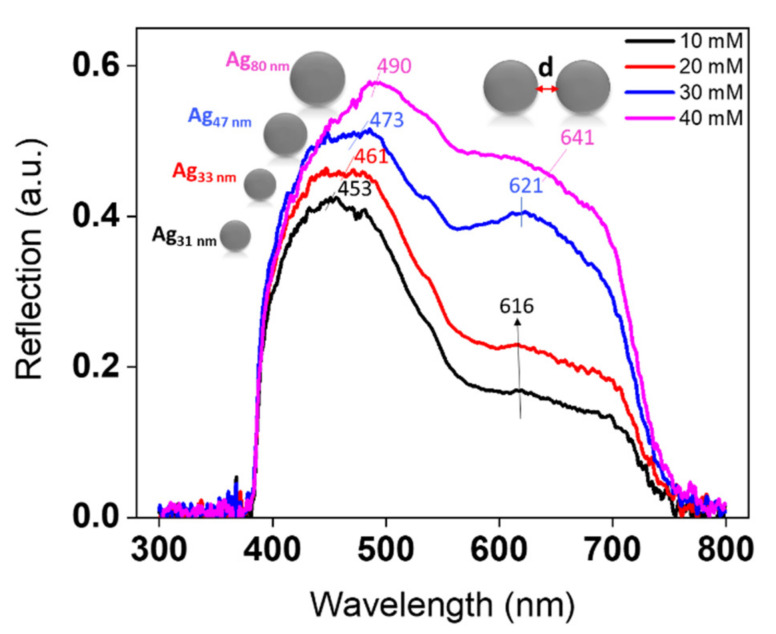
Spectral evolution of reflection of AgNPs/PMMA nanocomposites upon varying the concentration of Ag precursor.

The optical properties of the synthesized substrates were analyzed through micro-extinction (in reflection mode) and ellipsometric optical measurements. Figure 7 demonstrates the reflection spectra for AgNPs/PMMA at different concentrations. Herein, the change in the structural features of AgNPs confirms that the optical properties can be tuned by varying the concentration. The most conspicuous observation to emerge from the spectroscopic data was the ability of this technique to give such an optical response on monolayers of NPs on the surface. The spectrum of 10-mM AgNPs shows a peak at 453 nm. Similarly, the spectra of 20- and 30-mM AgNPs result in a slight red-shift to 461 and 473 nm, respectively. This slight red-shift is related to changes in the sizes of AgNPs from 31 to 47 nm. With a further increase in the concentration of Ag precursor to 40 mM, the position of this peak is continuously red-shifted into 490 nm. The position of the peak at ~490 nm is mainly attributed to the optical response of 80-nm AgNPs. As outlined in the literature review, the extinction spectra of 50 nm AgNPs dispersed in water showed a resonance peak at ~430 nm [38]. Changing the medium near the NPs’ surface from water to PMMA should induce a ~30 nm shift in the optical properties, due to an increase in the refractive index (RI) of the surrounding medium. Increasing the size of AgNPs to 80 nm should also exhibit a continuous red-shift of ~30 nm [39].

Otherwise, a pronounced change in plasmonic properties was observed when considerably increasing the concentration of Ag precursor to 30 and 40 mM, where the additional second peak appeared clearly. This peak was red-shifted from 616 to 641 nm as the concentration of Ag precursor increased from 10 to 40 mM. This peak refers to a coupling phenomenon, and its position is highly influenced by the gap distances separating the AgNPs. The two reasons for not observing a clear response of the second peak at low concentrations are that (1) there are not enough NPs viewed by the microscope objectives on the excited surface, and (2) the AgNPs are separated by large gaps. It is expected that the number of resonance peaks increases as the symmetry of the structure decreases. As clarified in Figure 6, the VIPS approach enables the fabrication of AgNPs in a nearly isotropic environment, thus impeding the possibility of polarizing electrons in more than one way. Furthermore, enlarging metal nanostructures caused only slight shifts of plasmonic properties, as stated by Paramelle et al. [39]. The plasmonic peak could only be tuned to ~ 500 nm, even if the size of the AgNPs reached 100 nm [39]. Thus, at 40 mM, this remarkable red-shift to 641 nm is due to the existence of a large number of hotspots that yield a high electromagnetic enhancement between closely separated 80-nm AgNPs.

Based on discrete dipole approximation (DDA) theoretical simulations, the absorption optical response of 80 nm nanosphere dimers, suspended in water with 10 nm gap distances, should induce a peak at ~560 nm [40]. The extent of this red-shift is maximized as the nanostructures become incredibly close to one another (<10 nm) [5]. With further decrease in gap distances and increase in the RI of the surrounding medium, when AgNPs dispersed in PMMA approach the surface of the Si substrate, the peak will be dramatically red-shifted to 600 nm and beyond. Figure 7 also indicates that the scattering intensity increased as the concentration of AgNPs changed from 10 to 40 mM. This can be regarded as an indicator of an increase in the density of the NPs on the surface of the substrate. It is relevant to note that the micro-extinction optical technique is not suitable for nanostructures with dimensions of less than ~30 nm. Ordinarily, for small particles with a size of <30 nm, absorption has ascendancy over scattering, thus making it complicated in detecting an optical response directly [41].

Recently, it was investigated that ellipsometry can be used for interpreting the optical spectra of the assemblies of anisotropic gold nanocubes (AuNC) on opaque substrate surfaces [30,33]. Ellipsometric theoretical calculations, based on physical modelling, were performed on AgNP samples in order to acquire a deep knowledge about their optical features. The model proposed previously by Rana et al. was improved by adding a third oscillator for NPs. This model system was chosen because it assumes that the mixture is composed of three materials (inclusions and host) playing asymmetric roles. Furthermore, we selected it in order to consider the spherical inclusions, with high volume fractions and high interactions between one another. For a detailed review of the technique and model used, see our previous papers [30,33].

**Figure 8:**
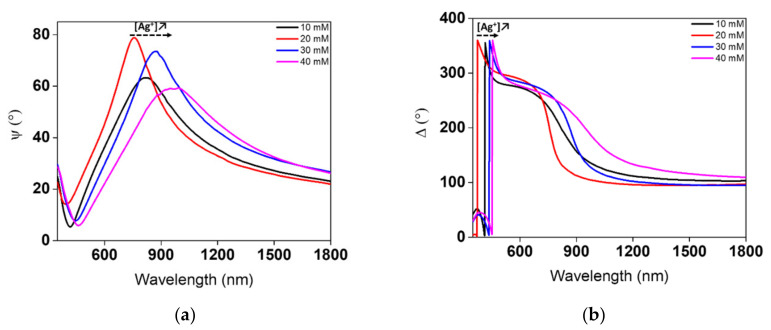
Variations of experimental ellipsometric angles (**a**) Ψ and (**b**) Δ in AgNPs/PMMA film/c-Si structures, as functions of the concentration of the Ag precursor.

As noted in Figure 8, experimental ellipsometric angles (Ψ and Δ) are characterized by a marked red-shift in positions as concentration is increased. However, a slight shift in Δ angles occurs, thus denoting that the thickness of the substrates is slightly shifted. The Δ angle is usually more sensitive to the thickness of the composite layer than the Ψ angle. The broadness and shift of the Ψ angle with increased concentration is widely affected by the increase in the ratio of the number of AgNPs to their corresponding thickness. As expected, there are some discrepancies in the sample prepared at 10 mM. The ellipsometric angles at 10 mM lie between the ones prepared at 20 and 30 mM. This apparent lack of correlation can be justified by the high nonuniformity of the sample over the area of the excitation beam. A closer inspection for this sample at high magnification reveals that its surfaces are full of PMMA aggregates in different regions. This lack of agreement could also be linked to the low-volume fraction of AgNPs on the excited surface. Therefore, a low number of photons will be collected when exciting the weakly interacting plasmonic NPs with the matrix layer. The high surface roughness of the sample prepared at 10 mM could have influenced the results obtained. As is well known, ellipsometric measurements and modelling are extremely hard to conduct when light scattering by surface roughness reduces the reflected light intensity severely.

**Figure A5:**
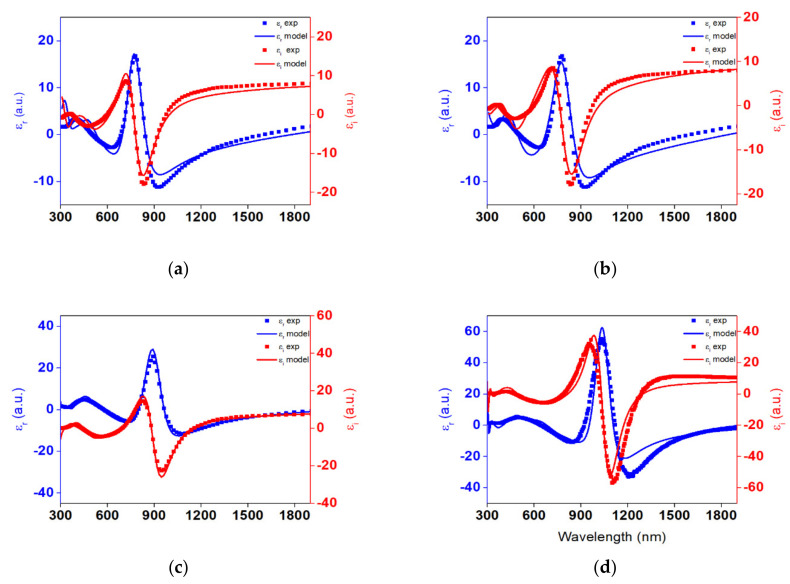
Comparison between experimental and fitted pseudo-imaginary and real dielectric functions (ε_i_, ε_r_) of AgNPs/PMMA film/c-Si structurea at different concentrations of Ag precursor: (**a**) 10; (**b**) 20; (**c**) 30; and (**d**) 40 mM.

**Figure A6:**
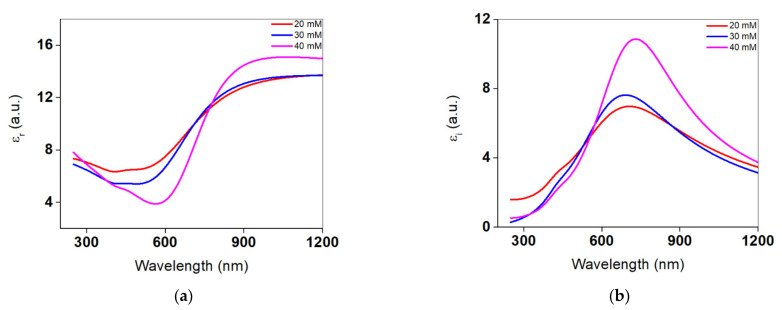
Dielectric functions ((**a**) Ԑ_r_ and (**b**) Ԑ_i_) of Ag/PMMA samples at different concentrations.

**Figure A7:**
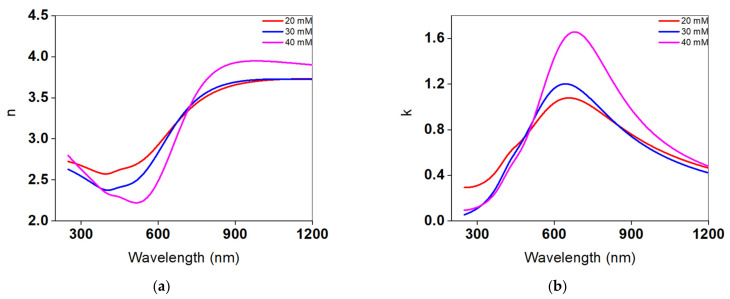
Refractive index ((**a**) n) and extinction coefficients ((**b**) k) of AgNPs/PMMA samples at different concentrations.

In Figure A5, to some extent, the experimental pseudo-dielectric functions of AgNPs/PMMA film/c-Si structures match fairly well with the fitted ones, and further emphasize the validity of our model. Slight disagreement is evident in some regions, since size distribution is not counted in our modelling. In our view, modelling pseudo-dielectric functions represents a good initial step toward determining the absorption coefficient (α) of Ag/PMMA samples, as mentioned in the insets of Figure 6. The dielectric functions (Ԑ_r_ and Ԑ_i_), refractive index (n), and extinction coefficient (k) can be found in the Appendix (Figure A6 and Figure A7). The insets in Figure 6 reveal that two plasmonic bands are observed at each concentration, and their maximum is highly red-shifted when increasing the concentration.

The maximum wavelength λmax of the first plasmonic band is progressively red-shifted from 418 to 430 nm with increasing concentration. The observed shift can be interpreted as being a result of the increase in size of AgNPs from 7 to 22 nm. These peaks are also visible in the Mie calculations, and have been assigned to the optical responses of small AgNPs through quantitative analysis based on calculations of Mie extinction cross-sections. [42,43]

Further shifts in the λmax of the second plasmonic band, from 595 to 645 nm, with preferential increase in intensity, are related to the coupling between AgNPs that are affected by the RI of PMMA. The increase in intensity is associated with the huge number of AgNPs that exist on the substrate surface. It is apparent from Figure 7 that the relative positions of the LSPR bands of the second peak are in good agreement with the ellipsometric results.

**Figure 9:**
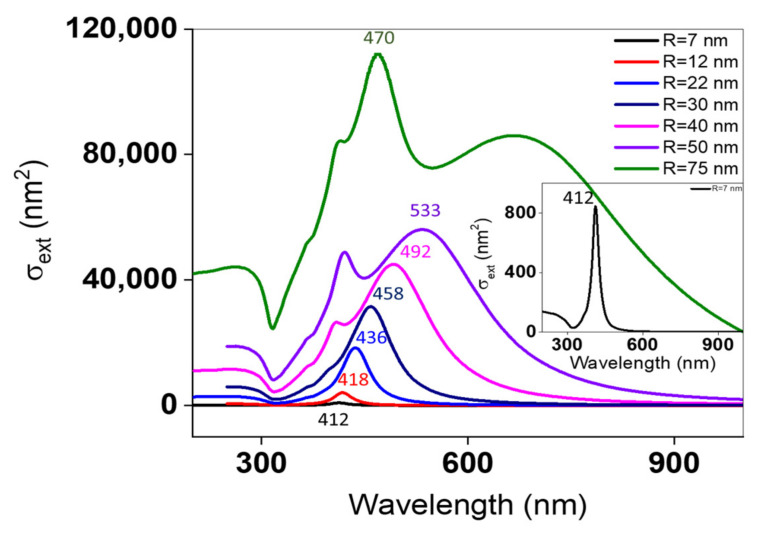
Mie theory calculations for the extinction cross-sections of different sizes of AgNPs suspended in a highly porous PMMA.

Theoretical spectra calculated using Mie theory for a homogeneous nanosphere are outlined in Figure 9, in order to determine the sum of the scattering and absorption cross-sections of spherical NPs. Simulations were carried out on single AgNPs, of different sizes, dispersed in a highly porous PMMA. Theoretical simulations of AgNPs at a fixed RI of PMMA ~1.25, with the radius varying from 7 to 50 nm, predict a red-shift of the LSPR wavelength of major dipolar peaks with an increased extinction efficiency. In general, large nanostructures eventually lead to the appearance of multiple SP modes other than the dipole modes.

The characteristic peaks for ~30 nm AgNPs at 20 mM are too close to distinguish between experimental results (453–461 nm) and theoretical simulation spectra (458 nm). For R = 47 nm at 30 mM, it is expected theoretically to have a dipolar peak between 492–533 nm. However, µext results display a peak at 473 nm. This difference is due to the fact that in Mie simulations the information is derived from only one homogeneous nanosphere. AgNPs with R = 75 nm exhibit a dipolar peak at 470 nm. This value correlates favorably with µext results (~490 nm), and further supports our previous findings. [31,32,33] Presumably, some differences between simulations and µext results are likely due to ensemble averaging effects over many different sizes.

The micro-extinction technique is remarkably insensitive to the detection of small NPs (R < 30 nm) and quadrupolar modes of large NPs (R > 30 nm), whereas ellipsometry is sensitive to the most abundant network of NPs over all the surface.

Contrary to Mie simulations at fixed RI of PMMA, a micro-extinction setup can figure out the slight variations in the thickness and RI of PMMA as experimental parameters vary. Moreover, in µ-ext optical microscopy, the focal spot of an optical beam is analyzing a micrometric zone of NPs that represent many NPs. Taking that into account, our results should be validated by using two complementary techniques to get an overall optical response of all sizes of Ag samples. In this regard, a significant correlation was recognized between both experimental (µext) and theoretical (ellipsometric and Mie simulations, respectively) data.

### 3.2. SERS Analysis

**Figure 10:**
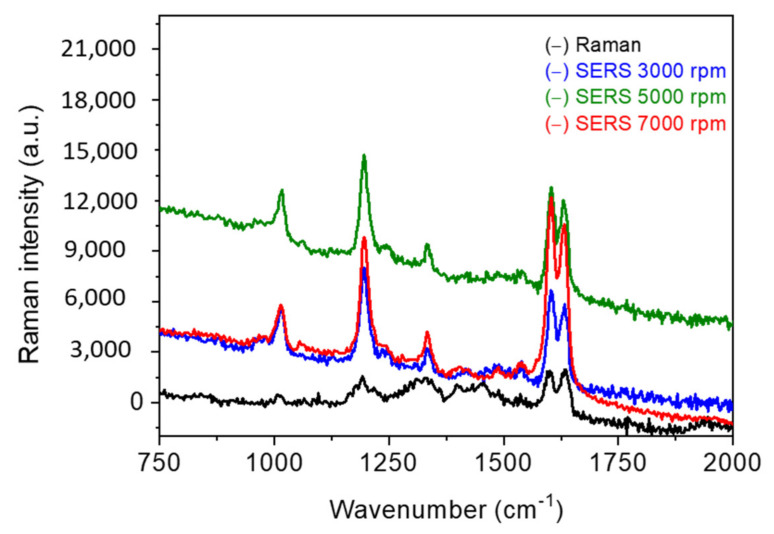
The Raman and SERS spectra of BPE at 10^−5^ M. Raman measurements were obtained with 10^−2^ M of BPE. Spectra were obtained at P = 5 mW over 5 s. Each curve is an average of 10 spectra collected from different positions on the substrate. All spectra were shifted vertically for the observation in all figures.

First, we studied the effect of spin-coating speed on the sensing properties of the obtained substrates. The results in Figure 10 show an enhancement of the SERS signal with increasing speed. To be precise, no major difference is observed between 3000 and 5000 rpm, which correlates strongly with the results shown in Figure 2, Figure 3, Figure 4. Below 7000 rpm, the samples revealed quite similar structural properties. The enhancement increase shown at higher speeds is attributed to the presence of higher numbers of nanoparticles on the substrate surface (Figure 2), which is related to closely separated nano-objects. As a result, more hotspots might exist at higher speeds, allowing for better SERS enhancement. To go further in the evaluation of the sensing features, we then performed SERS for the samples shown in Figure 6. Here, we showed that change in the structural and optical properties of AgNPs when silver precursor concentration was varied.

**Figure 11:**
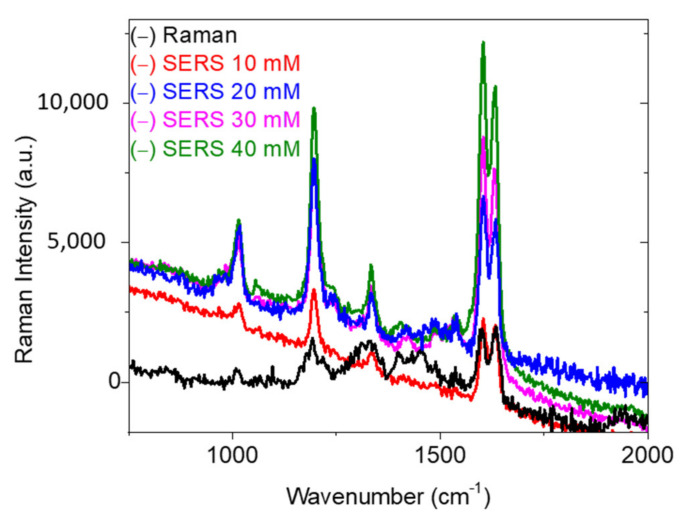
The Raman and SERS spectra of BPE at 10^−10^ M for AgNPs prepared with different silver precursor concentrations. Raman measurements were obtained with 10^−2^ M of BPE. Spectra were obtained at P = 5 mW over 5 s. Each curve is an average of 10 spectra collected from different positions on the substrate.

Similarly, Figure 11 also highlights noticeable results by showing an enhancement of the SERS signal with increasing precursor concentration. According to Figure 6, the number of AgNPs increases with Ag precursor concentration. Moreover, the second plasmon peak—which is linked to coupling—shifted from ~600 nm (10 mM) to 645 nm (40 mM). This latter figure is quite close to the excitation wavelength (633 nm) used for the experiments. This feature (λ_excitation_-λ_max_ = 0) is crucial, and plays a major role in enhancing the Raman intensity. For this reason, we might obtain a higher SERS enhancement factor for AgNPs prepared with larger precursor amounts. It is noteworthy that, in this section, we were able to obtain a SERS signal using a 1-s acquisition time. Therefore, we tried to decrease the BPE concentration to 10^−10^ M, and it was possible to obtain a SERS signal at this concentration value. To be in the same experimental conditions in Raman and SERS, which is crucial for any enhancement factor calculation, we performed measurements of both signals for 5 seconds. Based on literature [31,32] using the same type of PMMA nanocomposites for SERS, we related the high sensitivity of the samples to the repulsive interaction that exists between PMMA and nanoparticles. Thus, by pushing the BPE, PMMA consequently directs it onto the nanoparticles’ surface. As a result, the target molecules will be concentrated in the SERS focal volume, which highly increases the SERS intensity.

Precisely, BPE molecules adsorb to the metallic nanoparticles through the nitrogen groups, which makes pyridine derivatives very good candidates for SERS measurements. Thus, since the interaction takes place on nitrogen, which has more affinity for the metal than for PMMA, the BPE will be self-driven towards the plasmons. This finding has been reported in our previous studies [31,32,33].

Then, we employed the peak at 1605 cm^−1^ to estimate the SEF through the following equation:

SEF = (I_SERS_ × N_normal_)/(I_normal_ × N_SERS_)

where I_SERS_ and I_normal_ are the intensities of the same band for the SERS and normal Raman spectra, respectively. N_SERS_ is the number of molecules probed in SERS, and N_normal_ is the number of molecules excited in classical Raman. SEF corresponds to the SERS intensity of one molecule divided by the Raman intensity of one molecule without the SERS substrate, and can be seen as an absolute enhancement factor of the scattering cross-section of the test molecule. SEF calculation requires the excitation and collection volumes in the solution to be known. Knowing the probe molecule concentration, the N_normal_ can then be estimated. N_SERS_ can be obtained from knowledge of the active surface area of the substrate that is being probed, the footprint of an adsorbed molecule, and the surface coverage.

The focal volume of our Raman system is 11 fl. The SERS confocal volume (SCV) corresponds to 1.5 × 10^−3^ fl, considering the size of our nanoparticles. SCV was calculated using the following equation:

SCV = S × h

where S and h are the excited surface and height, respectively.

S = πr^2^, and h corresponds to beam penetration depth,

where r is the beam radius, and is calculated based on the excitation wavelength value (632.8 nm). Then, S is multiplied by the percentage of surface coverage by the nanoparticles. Finally, we multiply S by the number of nanoparticles determined after calculation of the surface of one NP, based on SEM images.

These volumes allow the detection of 3 × 10^7^ molecules in Raman, and 5 molecules in SERS, for a concentration of 10^−10^ M. At 1605 cm^−1^, I_SERS_ and I_normal_ correspond to 12,000 and 2100, respectively. From there, we obtained EF 3 × 10^10^ for the 10-mM sample, while 10^13^, 5 × 10^14^, and 10^15^ were determined for 20, 30, and 40 mM, respectively. The obtained high values of EF can be attributed to the hydrophobic features of the PMMA layer embedded with the AgNPs. This finding was reported and discussed in detail in our previous studies [30,31,32,33]. Indeed, at high BPE concentrations, multiple layers of molecules could be detected. Therefore, decreasing the concentration might decrease the amount of BPE adsorbed on the Au nanoparticles. Furthermore, it can be expected that we tend toward the presence of one layer of molecules close to the nanoparticles’ surface, which causes the Raman exaltation and then leads to this increase in the EF.

## 4. Conclusions

We have obtained comprehensive results demonstrating the value of the VIPS approach in conducting efficient SERS platforms (detection of five molecules) with controlled structural and sensing properties. The significance of our work lies in fabricating large-scale SERS-active opaque substrates, with high density of hotspots, yielding huge enhancements. Better surface roughness and overall bulk flatness, homogeneity of refractive index, and absence of secondary back reflection make silicon and opaque substrates greater for optical characterizations and applications. Correlating the structural properties of AgNPs with SERS enhancements has shown an enormous benefit in advancing both the perception of the fundamental mechanisms of SERS effects, and the strategies used to control the assembled NPs for efficient sensing applications.

This study could further provide the framework for triggering the formation of advanced, multifunctional hybrid materials such as Ag–Au organized inside PMMA nanoholes. This could conceivably lead to promising sensing features.

## Figures and Tables

**Table 1:** Average diameters of AgNPs (D1 and D2) at different concentrations.

Concentration of Ag (mM)	D1 (nm)	D2 (nm)
10	<7	31
20	7	27–33
30	7–13	43–47
40	12–22	80

## Data Availability

The data presented in this study are available on request from the corresponding author.
